# Clinical assessment of adenosine stress and rest cardiac magnetic resonance T1 mapping for detecting ischemic and infarcted myocardium

**DOI:** 10.1038/s41598-020-71722-3

**Published:** 2020-09-07

**Authors:** Sirilak Yimcharoen, Shuo Zhang, Yodying Kaolawanich, Prajak Tanapibunpon, Rungroj Krittayaphong

**Affiliations:** 1 Division of Cardiology, Department of Medicine, Faculty of Medicine Siriraj Hospital, Mahidol University, 2 Wanglang Road, Bangkoknoi, Bangkok, 10700 Thailand; 2 Philips Healthcare, Hamburg, Germany; 3 Faculty of Medicine Siriraj Hospital, Her Majesty Cardiac Center, Mahidol University, Bangkok, Thailand

**Keywords:** Cardiology, Imaging

## Abstract

Cardiac magnetic resonance (CMR) spin-lattice relaxation time (T1) may be influenced by pathologic conditions due to changes in myocardial water content. We aimed to validate the principle and investigate T1 mapping at rest and adenosine stress to differentiate ischemic and infarcted myocardium from controls. Patients with suspected coronary artery disease who underwent CMR were prospectively recruited. Native rest and adenosine stress T1 maps were obtained using standard modified Look-Locker Inversion-Recovery technique. Among 181 patients included, T1 values were measured from three groups. In the control group, 72 patients showed myocardium with a T1 profile of 1,039 ± 75 ms at rest and a significant increase during stress (4.79 ± 3.14%, *p* < 0.001). While the ischemic (51 patients) and infarcted (58 patients) groups showed elevated resting T1 compared to controls (1,040 ± 90 ms for ischemic; 1,239 ± 121 ms for infarcted, *p* < 0.001), neither of which presented significant T1 reactivity (1.38 ± 3.02% for ischemic; 1.55 ± 5.25% for infarcted). We concluded that adenosine stress and rest T1 mapping may be useful to differentiate normal, ischemic and infarcted myocardium.

## Introduction

Coronary artery disease (CAD) is one of the major health problems that creates economic burden in both developed and developing countries^1^. Although invasive coronary angiogram is the gold standard for diagnosis of CAD, report from national registry of United States of America revealed only 41% obstructive coronary lesion of patients who were scheduled for invasive coronary angiogram with clinical indication^2^. To avoid unnecessary invasive procedures, many non-invasive investigations have been proposed as a gatekeeper before coronary angiogram^3,4^. Practice guidelines recommend, in order to benefit from revascularization, patients should have at least 10% of ischemic myocardium^5^.

Cardiac magnetic resonance (CMR) is one of the standard imaging techniques for assessment of myocardial ischemia and scar^6^. Conventional CMR performed during stress perfusion and late gadolinium enhancement (LGE) differentiates myocardial area of ischemia and scar based on perfusion defect revealed by contrast bolus during adenosine stress and hyperintensity at the late stage after contrast injection, respectively^7^. Recently, CMR T1 mapping and extracellular volume (ECV) assessment has shown an emerging role for myocardial tissue characterization that offers possibility in diagnosis of various types of cardiomyopathy and infiltrative disease^8,9^. For example, in chronic infarction an increase in the native T1 value has been revealed due to expansion of ECV caused by replacement fibrosis within both infarct territory and remote myocardium^10–13^. As the measured quantitative values directly reflect intrinsic myocardial properties in both healthy and diseased conditions^9,11,14^, the technique may potentially help to indirectly estimate myocardial blood volume (MBV) and myocardial water content^10,15^. Recently a proof-of-principle exploiting rest and stress T1 mapping for detection of increased MBV induced by coronary vasodilatation in patients with severe aortic stenosis and CAD was successfully demonstrated in a small number of patients^16,17^.

**Figure 1:**
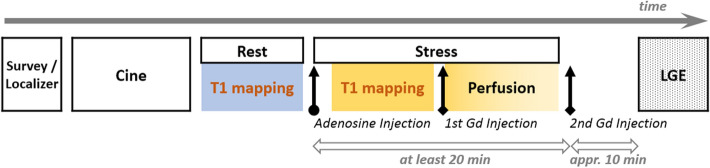
Cardiac magnetic resonance (CMR) examination paradigm for clinical assessment of coronary artery disease (CAD). Scan protocol was shown from survey (left) to LGE (right) in time sequence. Details see text. LGE = late-gadolinium enhancement, Gd = gadolinium.

The aim of this study was to validate and assess this principle of native CMR T1 mapping at rest and during adenosine stress (scan protocol in Fig. 1 and imaging sequence parameters in Table 1) to differentiate control, ischemic, and infarcted myocardium in suspected CAD patients.

**Table 1:** CMR imaging parameters in clinical assessment of suspected CAD patients.

	Cine	Perfusion	LGE	T1 mapping
FoV (mm^2^)	256 × 240	270 × 330	384 × 303	340 × 290
Pixel size (mm^2^)	1.5 × 1.2	1.7 × 1.9	1.5 × 1.26	1.8 × 1.8
Slice thickness (mm)	8	8	8	8
TR/TE (ms)	3.7/1.8	9/1.7	4.1/1.25	2.8/1.38
Flip angle (°)	60	15	15	30
Min. TI (ms)	–	–	–	93.4
Motion compensation	Retrospective ECG-gating, breathhold	ECG-triggering, breathhold	ECG-triggering, breathhold	ECG-triggering, breathhold

*FoV* field of view, *TR *repetition time, *TE *echo time, *TI* inversion time, *LGE* late-gadolinium enhancement.

## Results

### Study participants and T1 results

**Figure 2:**
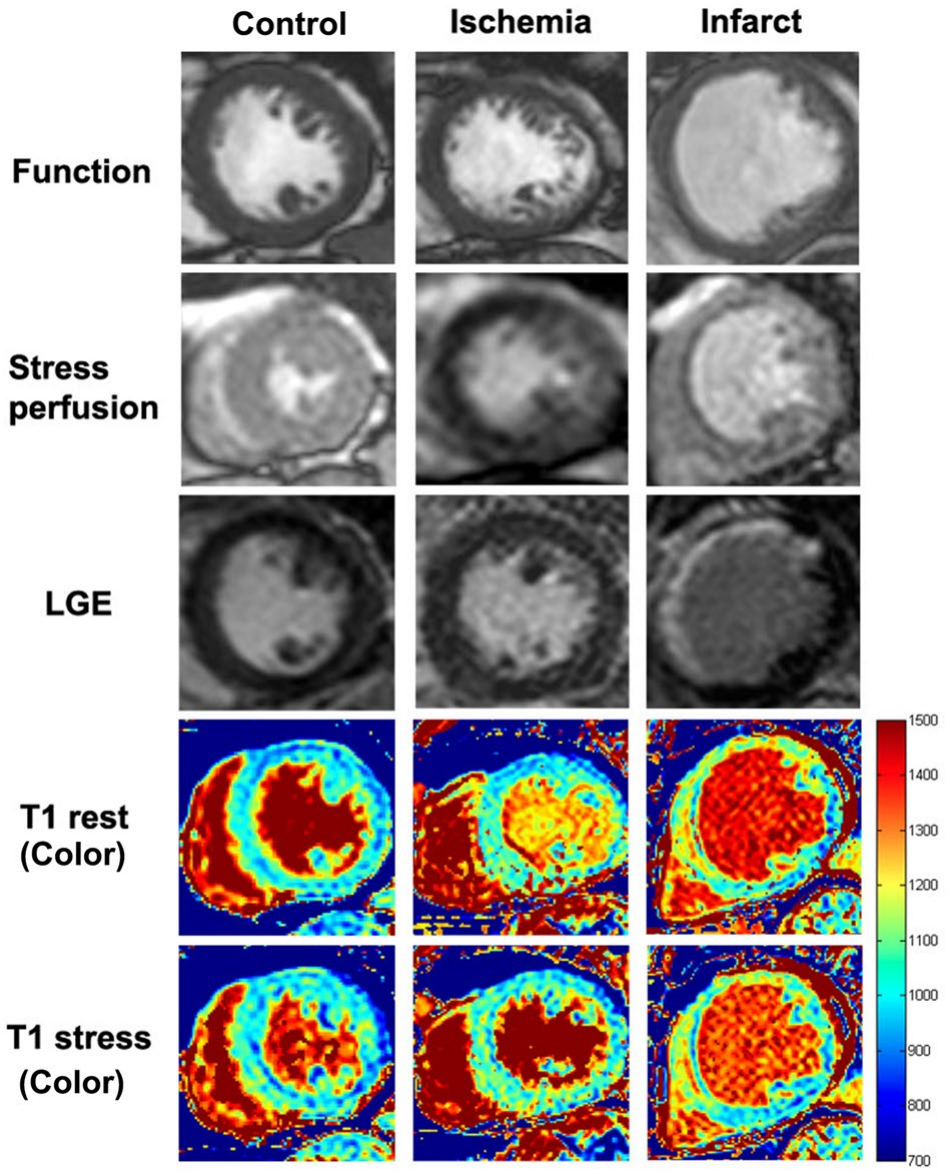
CMR images of function (top panel), stress perfusion (2nd panel), LGE (3rd panel), color map of T1 at rest and stress (4th and 5th panel). The control group (left) showed normal perfusion with no late-gadolinium enhancement (LGE). The ischemic patient (middle) showed first-pass perfusion defect at the septal wall without LGE. The infarcted patient (right) showed first-pass perfusion defect and positive LGE at the anterior and septal walls.

**Figure 3:**
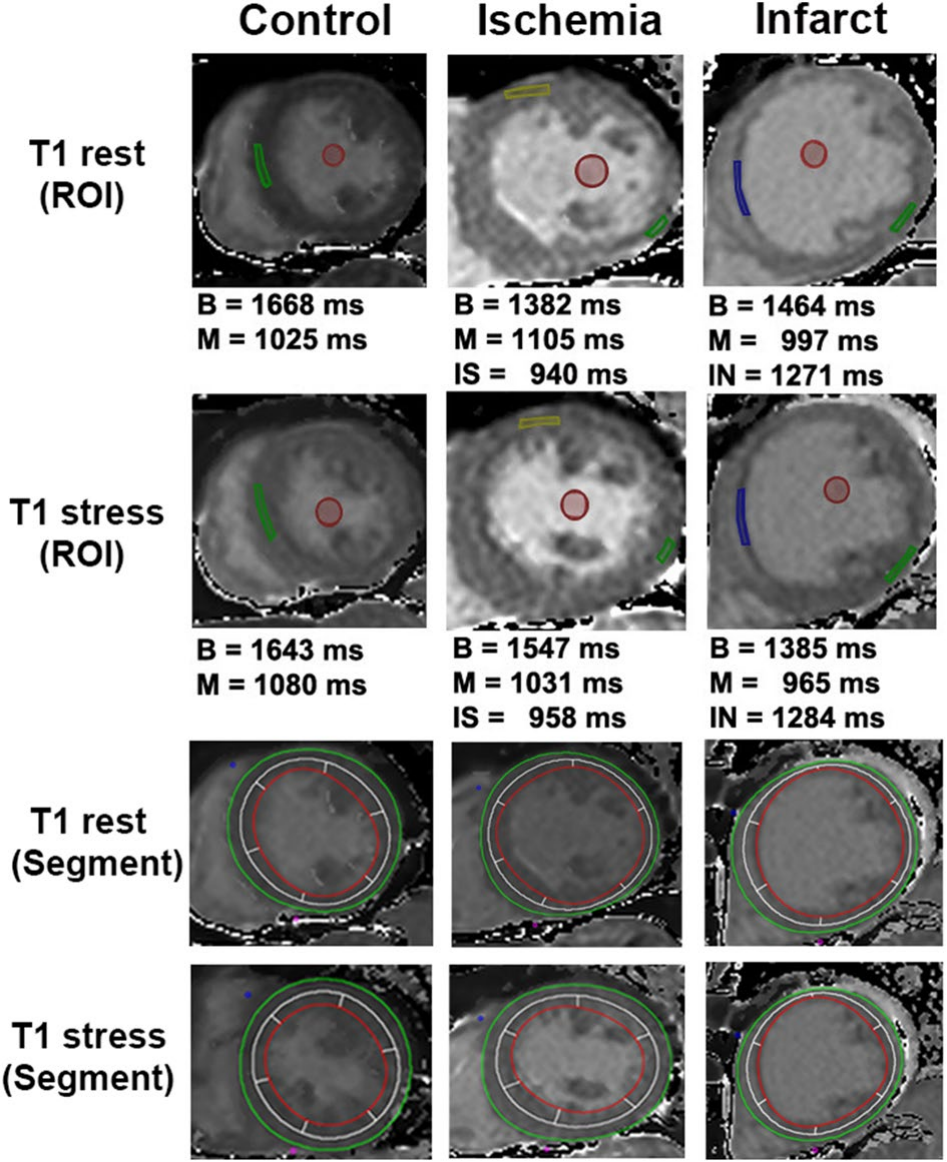
Definition of region of interest (1st and 2nd panel) and subendocardial contour (3rd and 4th panel) on myocardial T1 maps at rest and stress for group analysis.

One hundred eight-one suspected CAD patients were enrolled, and 95 (52.5%) were male. Patients were divided into 3 groups according to the results of first-pass perfusion and LGE imaging. Details were as follows: (1) *control group* was defined as negative in both first-pass perfusion and LGE (72 cases); (2) *ischemic group* was defined as the presence of perfusion defect without LGE (51 cases); and, (3) *infarcted group* was defined as the presence of LGE (58 cases). Characteristics of these 3 groups, including CMR findings, are shown in Table 2, while typical CMR examples of function, stress perfusion, LGE, rest and stress T1 mapping are shown in Fig. 2. Region of interest (ROI) and subendocardial contour were manually defined on the T1 maps for group analysis including per-segment analysis (Fig. 3).

**Table 2:** Baseline demographic and clinical characteristics of the study population.

Characteristics	Normal (n = 72)	Ischemia (n = 51)	Infarct (n = 58)	*p*-value
Male gender	25 (34.7%)	30 (58.8%)	40 (69.0%)	< *0.001*
Age (years)	70.89 ± 10.45	71.20 ± 11.65	66.84 ± 12.36	0.074
BMI (kg/m^2^)	25.29 ± 3.66	25.47 ± 3.97	25.63 ± 6.99	0.929
HR (beats/min)	71.97 ± 14.27	73.98 ± 12.36	72.72 ± 13.15	0.715
SBP (mmHg)	136.51 ± 21.17	136.65 ± 17.15	125.69 ± 20.02	*0.003*
DBP (mmHg)	75.32 ± 12.86	75.10 ± 11.30	74.16 ± 14.44	0.871
**Risk factor**				
Smoking	0 (0.0%)	0 (0.0%)	0 (0.0%)	–
Hypercholesterolemia	35 (48.6%)	31 (60.8%)	30 (51.7%)	0.399
Diabetes mellitus	27 (37.5%)	28 (54.9%)	28 (48.3%)	0.146
Hypertension	60 (83.3%)	40 (78.4%)	43 (74.1%)	0.438
Family history of IHD	0 (0.0%)	0 (0.0%)	0 (0.0%)	–
**Medications**				
Beta-blocker	34 (47.2%)	36 (70.6%)	47 (81.0%)	< *0.001*
CCB	26 (36.1%)	19 (37.3%)	7 (12.1%)	*0.003*
Nitrates	5 (6.9%)	16 (31.4%)	8 (13.8%)	*0.001*
Aspirin	28 (38.9%)	45 (88.2%)	52 (89.7%)	< *0.001*
ACEI	7 (9.7%)	4 (7.8%)	17 (29.3%)	*0.002*
ARB	21 (29.2%)	12 (23.5%)	38 (34.5%)	0.455
Statin	40 (55.6%)	41 (80.4%)	48 (82.8%)	*0.001*
**CMR data**				
LVEF (%)	74.19 ± 9.43	64.49 ± 14.32	48.95 ± 15.78	< *0.001*
Left ventricular mass (g)	78.37 ± 22.38	88.81 ± 22.15	101.43 ± 36.93	< *0.001*
Cardiac output (L/min)	5.9 ± 1.73	5.9 ± 1.33	5.69 ± 2.43	0.796
Stroke volume (ml)	82.18 ± 18.04	80.42 ± 15.76	78.89 ± 29.02	0.689

Data presented in mean ± standard deviation.

A *p*-value < 0.05 indicates statistical significance (italics).

*BMI* body mass index, *HR* heart rate, *SBP* systolic blood pressure, *DBP* diastolic blood pressure, *IHD* ischemic heart disease, *CCB* calcium channel blocker, *ACEI* angiotensin converting enzyme inhibitor, *ARB* angiotensin receptor blocker, *CMR* cardiac magnetic resonance, *LVEF* left ventricular ejection fraction.

### Group analysis of CAD patients

**Figure 4:**
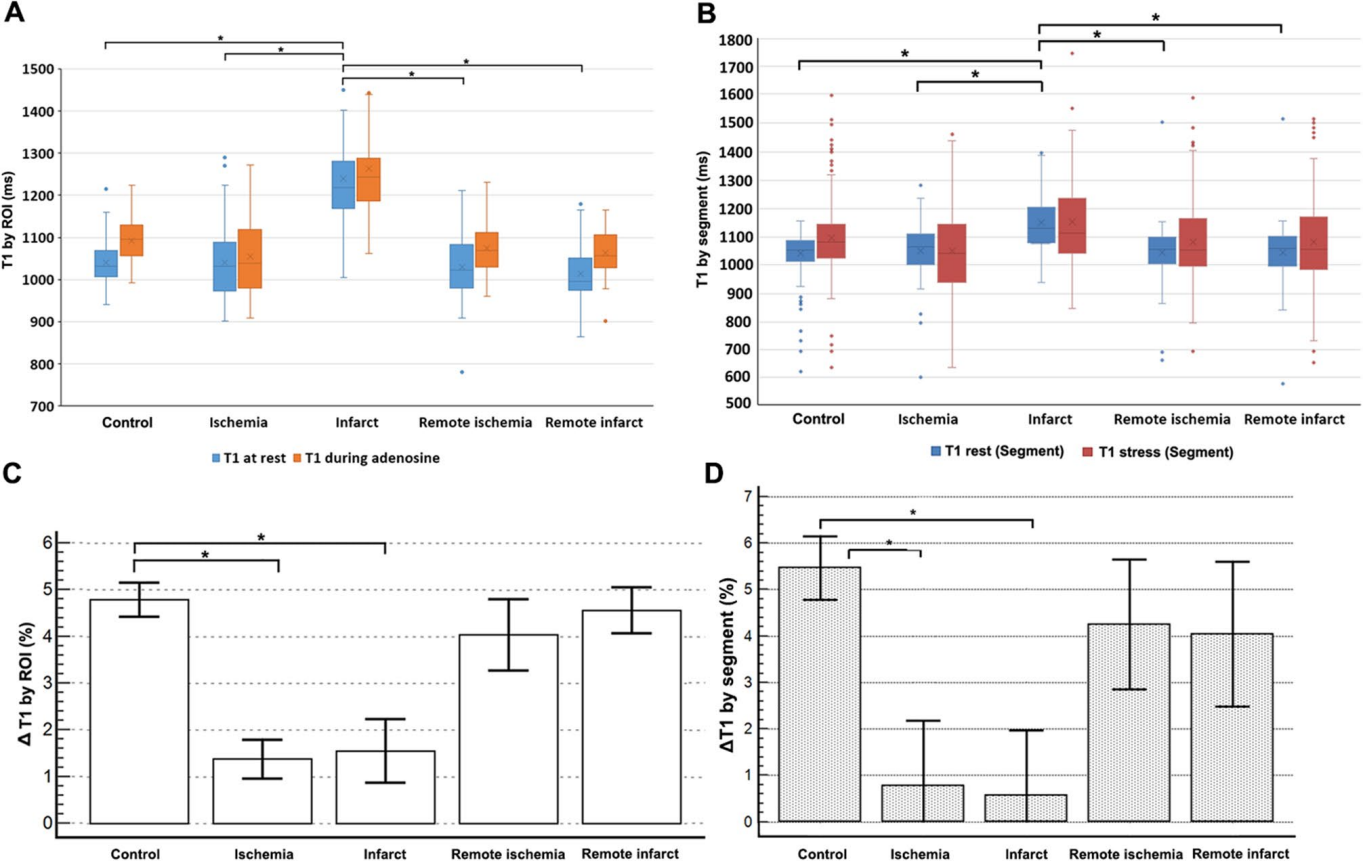
Myocardial T1 at rest and during adenosine stress in ROI (**A** and **C**) and per-segment (**B** and **D**) analysis. Both absolute values (T1 in ms, **A** and **B**) and percentage differences (ΔT1 in %, **C** and **D**) were shown for different groups. *p < 0.001.

In the control group, the mean T1 value at rest via ROI analysis was 1,039 ± 75 ms (ms). During adenosine stress the mean T1 significantly increased to 1,092 ± 50 ms (*p* < 0.001), with an absolute difference ΔT1 of 52 ± 35 ms and percentage change (%) of 4.79 ± 3.14, both of which were significantly greater than those observed in the ischemia and infarct groups (Table 3 and Fig. 4).

**Table 3:** Myocardial T1 characteristics from different groups of the study population with suspected CAD.

	Control (n = 72)	Ischemia (n = 51)	Infarct (n = 58)	Remote ischemia (n = 51)	Remote infarct (n = 58)
**ROI analysis**					
T1 rest (ms)	1,039.59 ± 75.63	1,040.13 ± 90.02	1,239.04 ± 121.39^a,b,c,d^	1,030.12 ± 75.63	1,013.54 ± 59.90
T1 adenosine stress (ms)	1,092.26 ± 50.63	1,055.20 ± 91.01^e^	1,262.80 ± 162.85^a,b,c,d^	1,073.81 ± 59.38	1,062.25 ± 50.48^f^
ΔT1 (%)	4.79 ± 3.14	1.38 ± 3.02^e^	1.55 ± 5.25^a^	4.04 ± 5.49	4.57 ± 3.80
**Per-segment analysis**					
T1 rest (ms)	1,033.71 ± 76.4	1,040.68 ± 107.00	1,142.16 ± 99.58^a,b,c,d^	1,035.89 ± 94.59	1,036.59 ± 89.32
T1 adenosine stress (ms)	1,087.77 ± 130.64	1,042.58 ± 153.06	1,145.97 ± 164.49^a,b,c,d^	1,072.29 ± 145.87	1,072.86 ± 175.51
ΔT1 (%)	5.46 ± 12.29	0.78 ± 14.98^e^	0.58 ± 13.73^a^	4.25 ± 16.09	4.05 ± 18.16

Data presented in mean ± standard deviation.

A *p*-value < 0.05 indicates statistical significance.

*CMR* cardiac magnetic resonance, *SD* standard deviation; ms, milliseconds.

^a^Statistical significance between infarct and control.

^b^Statistical significance between infarct and ischemia.

^c^Statistical significance between infarct and remote ischemia.

^d^Statistical significance between infarct and remote infarct.

^e^Statistical significance between ischemia and control.

^f^Statistical significance between remote infarct and control.

The resting T1 in the infarcted myocardium/group was 1,239 ± 121 ms, which was significantly higher than that found in the control myocardium/group and ischemic myocardium/group (*p* < 0.001). At the same time, minimal T1 change was observed in the ischemia (ΔT1 = 15 ± 34 ms or 1.38 ± 3.02%) and infarct (ΔT1 = 23 ± 86 ms or 1.55 ± 5.25%) groups. The remote myocardium in both infarct and ischemia groups did not show significant difference compared to the control for both T1 rest and ΔT1 (in ms or %). Only remote infarcted myocardium revealed significantly lower T1 than the control during adenosine stress (1,062 ± 50 vs. 1,092 ± 51 ms, p = 0.006). Similar findings were also observed from the per-segment analysis comparing native T1 values at rest and during stress in different groups. Details are presented in Table 3 and Fig. 4.

**Figure 5:**
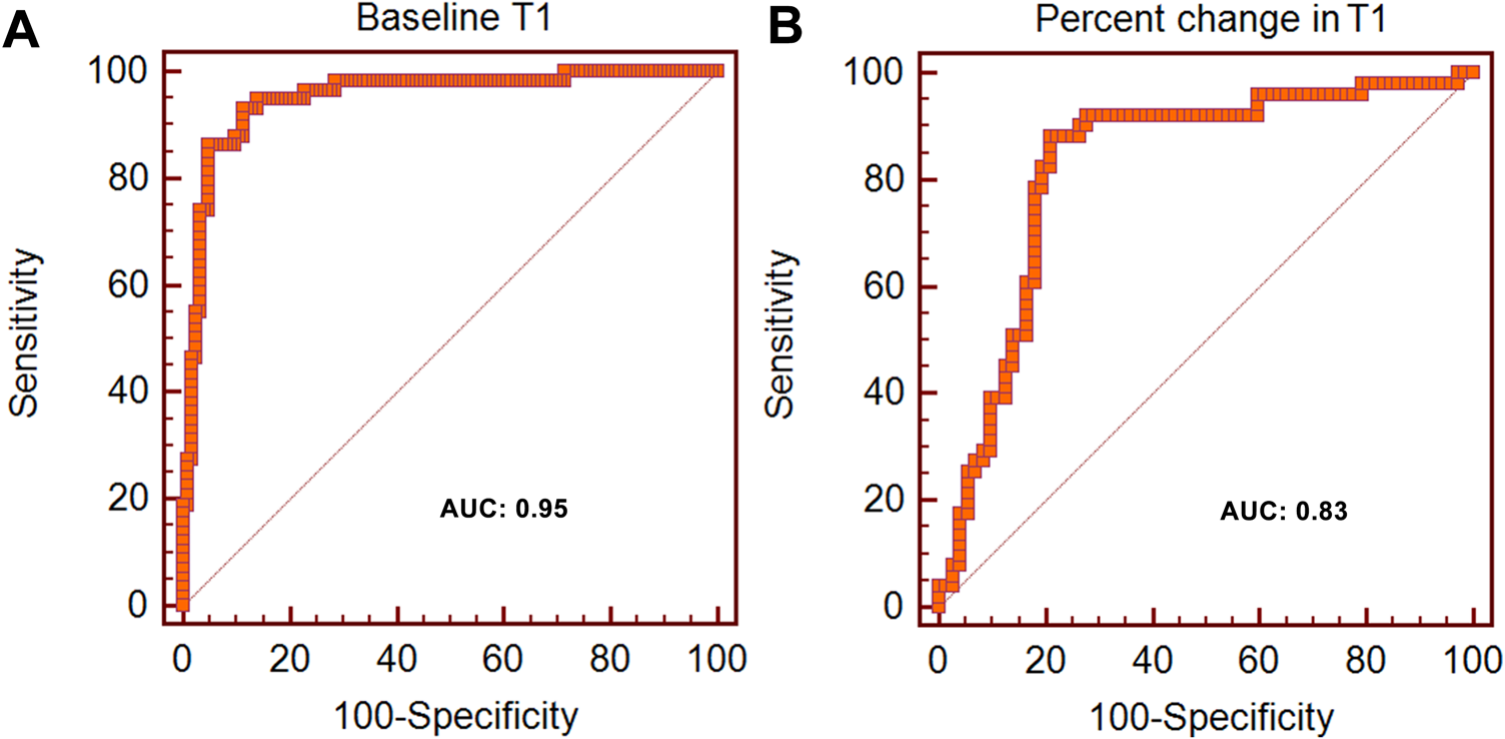
Receiver operating characteristic (ROC) curve analysis for distinguishing myocardial infarction (**A**) and ischemia (**B**) based on resting T1 and T1 reactivity (ΔT1 in %) during stress. AUC = Area under the curve.

Figure 5 shows that infarcted myocardium can be differentiated from control based on resting T1 with an area under the curve (AUC) of 0.95; ischemic myocardium can be differentiated from control based on T1 reactivity (ΔT1 in %) with AUC of 0.83.

## Discussion

This study has validated the principle of using native T1 at rest and during adenosine stress to differentiate area of myocardial ischemia and infarction from control in suspected coronary artery disease (CAD). The concept has been previously proposed by Liu et al. using the ShMOLLI sequence^16^, and is adopted in this work in a clinical setup with a larger cohort using the modified Look-Locker Inversion-Recovery (MOLLI) sequence, which is based on the same Look-Locker inversion recovery technique and has been widely investigated with well proven precision and repeatability^9,18–21^. The obtained result from the group analysis could serve as reference value for future studies applying the same approach. While an overall availability of the MOLLI sequence on clinical systems may facilitate clinical adoption, clinical trials with more strictly defined cohorts or in different populations are needed to and further verify the clinical findings.

Identification of myocardial ischemia and infarction is crucial in patients with suspected or known coronary artery disease in term of both diagnosis of ischemic heart disease, determination of myocardial area at risk and assessment of myocardial viability^3^. In chronic myocardial infarction, replacement of myocardial cells by scar or fibrosis has been observed with increased extracellular collagen, while area of acute and recent myocardial infarction contains increased intracellular water due to myocardial cell injury and swelling^12^. Both conditions lead to an increase of MBV and native T1. This has been reported in numerous studies in diagnosis and disease monitoring^9,11,13–15^. In the current study a native T1 at rest > 1,113 ms can well identify infarcted myocardium, which confirms its potential diagnostic role from the previously published studies, although the value varies among studies. For example, Liu, et al.^16^ reported a higher value (1,442 ± 84, vs. 1,239 ± 121 ms in the current study), which might be explained by the partial volume effect due to possible contamination of the blood pool for subendocardial scar^12^. In addition, different ages of infarction also contribute to such a variation, as scar tends to become denser over time^15^.

For T1 in the remote area in two CAD groups, although it revealed a lower adenosine reactivity compared to the control group, the result did not reach statistically significant difference, which has also been shown in the previous study^22^. This is different to the non-ischemic pathology, where normal-appearing myocardium may have an increased T1 due to subtle pathology^23^. The patients in this study, including both acute and chronic CAD, may or may not develop significant microfibrosis. We postulate that this explains the finding of the insignificant difference. Nevertheless, the lower T1 reactivity in the remote area may be associated with the blunt response to stress and indicate the subtle pathology that cannot be visualized in perfusion and LGE images. Moreover, in the control group a ROI was drawn in the septum, while in the infarct and ischemia groups it was placed in different segments to avoid perfusion defect or LGE. The inter-segment variation, as shown in multiple reports previously^24,25^ may also contribute to this finding here.

Ischemic myocardium at rest do not differ much from the controls in both structure and extracellular volume (ECV)^12^. It is, therefore, difficult to be differentiated by native T1 at rest, as shown in Table 3 and Fig. 3. Intravenous adenosine infusion leads to increased myocardial blood flow in the normal myocardium but not the ischemic or infarcted segment^26^. This difference of the resulting T1 reactivity could be used to distinguish these two groups, as demonstrated previously in the proof-of-concept study^22^ as well as the current validation study. While the blood flow increase distal to the stenotic coronary artery during adenosine stress is limited due to already compensated resistance vessels at rest, adenosine-induced vasodilation results in an increased MBV and thus a greater change of T1 increase in the normal coronary arteries (Table 3 and Fig. 3). A similar greater change in adenosine stress T1 has also been shown in a previous study based on AUC analysis compared to conventional adenosine stress CMR for diagnosis of obstructive CAD^22^.

Despite T1 during adenosine stress appeared significantly lower in the remote infarcted area than that in the control group, its changes or T1 reactivity was not significantly different among the three groups. The main reason could be explained by the fact that T1 at rest was slightly lower in both ischemic and infarct groups as well, as described above. This is also in consistency to the previous report^22^. Notably, for T1 mapping during adenosine stress in the present study, image acquisition using the MOLLI technique was performed 2 min after intravenous adenosine infusion, in contrast to 3 to 6 min in the previous reports^12,15^. As shown in the early study in human, adenosine is very short-acting medication and its intravenous infusion reached a maximal response on coronary blood flow at about 84 s after infusion start, with an appropriate dose of 140 µg/kg/min^26^. The shorter adenosine infusion time may be one of the reasons for the difference in T1 reactivity between our study and the previous one^16^. Another potential reason may be the study population difference. Liu et al. used healthy volunteers, whereas we had suspected CAD patients with negative adenosine stress CMR as controls. In addition, it could be partly explained by the sequence-specific phenomenon, as the applied MOLLI sequence in this work had a slightly different scheme comparing to ShMOLLI that was used previously^16^. In a different study a similar extent of T1 reactivity as ours was observed using the same MOLLI scheme^27^. Though this difference (ΔT1 in % = 4.79 ± 3.14 in the current study, vs. 6.2 ± 0.5% and 6.0 ± 4.2^16,17^) may be deemed as clinically insignificant, whether or not these factors might cause discrepancy in the exact T1 reactivity remains to be further investigated.

The per-segment analysis mitigating the T1 measurement bias has demonstrated a similar finding to the ROI analysis. Native T1 of the infarct segment was higher compared to that of the ischemia segment and of the control segment. It was, however, lower than that of the infarct group from the ROI analysis (Fig. 4). This could be explained by a mixture of the infarct and non-infarct areas in the same segment. T1 changes during stress also showed a similar trend as in the ROI analysis, where both the ischemic and infarcted segments exhibited a lower T1 reactivity compared to the control segments.

There are some limitations of this study. Firstly, no healthy volunteer data were included. Patients having clinical indication for CMR as suspected CAD without evidence of ischemia or infarction was used as the control group. It has been shown that patients with suspected CAD and negative adenosine CMR had an excellent prognosis with no major adverse cardiac event during follow up^28^. On the other hand, injection of contrast agent in healthy volunteers is not practical in a clinical setup and it is difficult to obtain the ethical approval. Although they did not show ischemia or scar, obstructive CAD might still be present due to false negative results from adenosine stress test. Even if obstructive CAD could be excluded patients might still have subtle myocardial pathologies with symptoms mimicking CAD. We did not have coronary angiography data to confirm CAD in those with ischemia or infarct. However, the T1 reactivity to adenosine stress in our study is consistent to the previous reports that had angiographic confirmation^22^. Secondly, T1 maps were obtained only from one short-axis slice at the mid LV level and no segment analysis was peformed in this study. However, this should not affect our result since we aimed to assess ischemic or infarcted myocardium compared to myocardium in the control group with T1 mapping. In addition, a second-based and heart rate-independent MOLLI scheme^21^ was not available on our system at the time of this study. We used the classic beat-based scheme and adapted it for different heartrate ranges to allow for sufficient longitudinal magnetization recovery between singal acquisitions that helped to compensate for heartrate variability. Despite this, a commercial implementation may be expected to fully address the issue in the future studies^20,29^ Further, T1 is well known to be technique and sequence dependent. This might limit the generalizability of the methodology. However, comparisons among different T1 mapping acquisition techniques demonstrated that myocardial T1 mapping is a powerful clinical tool for soft tissue characteristic classification in spite of the absence of established reference values^30^. Thirdly, we did not perform post-contrast T1 mapping and thus did not calculate extracellular volume (ECV). One potential source of improvement in the future studies would be estimation of ECV that reflects extracellular matrix comprising the interstitial space and intravascular space^31–33^. A direct estimation from the same MR scan may provide additional information helping to differentiate myocardium with subtle pathology from the normal one. However, as MBV indicates the blood volume within the microvascular circulation and thus a more comprehensive global marker for ischemia, it directly reflects the functional capacity of the coronary artery and provides the information of coronary stenosis severity^34^. Finally, yet importantly, the proposed algorithm was not tested in a foreign cohort for verification since it is beyond the scope of the study. This needs to be further investigated in the future prospective study.

In summary, the approach of using adenosine stress and rest CMR T1 mapping to differentiate myocardial tissue characteristics in CAD was validated. The obtained stress and rest T1 values in ischemic, infarcted and remote myocardium as well as controls can be used as reference, where future investigation is warranted to further derive the diagnostic algorithm and verify its clinical performance.

## Methods

### Patient selection

Patients aged years and above presenting symptoms suggestive of coronary artery disease (CAD) who underwent CMR during the study period from March to June 2017 were included. Patients having one or more of the following conditions were excluded: (1) clinical congestive heart failure; (2) pregnancy; (3) history of gadolinium allergy; (4) poor CMR image quality; (5) history of claustrophobia; and/or (6) inability to perform CMR due to implantation of a permanent pacemaker, internal defibrillator, or other metallic device. In addition, patients who had mixed infarction and ischemia were also excluded. Written informed consent was obtained from all patients before the MRI examination. The study protocol was approved by the Institutional Review Board of Siriraj Hospital and complied with the principles set forth in the Declaration of Helsinki (1964) and all of its subsequent amendments.

### CMR imaging

CMR imaging was performed on a clinical 1.5 T (T) system (Philips Achieva, Philip Healthcare, Best, The Netherlands) and included conventional cardiac function, myocardial perfusion, and late gadolinium enhancement (LGE) following the multi-slice multi-direction survey images. Functional cine was acquired by using a balanced turbo field-echo (bTFE) or steady-state free precession (bSSFP) sequence at two-, three-, and four-chamber views, as well as short-axis slices parallel to the mitral valve ring covering the left ventricle (LV) from base to apex.

T1 maps were acquired at rest and during peak adenosine stress (140 µg/kg/min, intravenously for 2 to 4 min) on the matching short-axis slice to cine at the midventricular level before gadolinium administration and first-pass perfusion. A standard ECG-triggered modified Look-Locker (MOLLI) sequence was applied within one single breathhold. The “native” 5-(3)-3 scheme was used including 5 acquisitions after the first inversion preparation, followed by a 3-heartbeat pause, and a second inversion pulse for the last 3 acquisitions^18,21,35^. This scheme was extended to compensate for heartrate dependence, particularly to ensure sufficient recovery of longitudinal magnetization during the pause interval between two acquisition blocks^20,21^.

First-pass perfusion imaging was performed, on three matching short-axis slices to cine at the apical, mid, and basal left ventricular levels, during peak adenosine stress with an intravenous bolus of gadolinium (0.05 mmol/kg; Magnevist, Bayer Schering Pharma, Berlin, Germany). LGE imaging was performed approximately 10 min after an additional bolus of gadolinium (0.1 mmol/kg), on matching short- and long-axis views to cine. The examination paradigm as well as scan protocol is presented in Fig. 1. Details of imaging sequence parameters are shown in Table 1.

### Image analysis

T1 maps were automatically generated on the MR system from the MOLLI series. All images including LV function, first-pass myocardial perfusion and LGE were analyzed offline using CVI42 software (Circular Cardiovascular Imaging Inc., Calgary, Canada). LV volume and mass was calculated and indexed according to the body surface area. Left ventricular ejection fraction (LVEF) was assessed using end-systolic and end-diastolic volume calculated from multiple-slice short-axis images. Myocardial perfusion defect and LGE images were analyzed by visual assessment. Myocardial ischemia was defined as the presence of perfusion delay at least 5 consecutive phases in 1 myocardial segment or more during first-pass perfusion^7^. Our earlier work demonstrated that using stress perfusion and viability images are accurate for the detection of significant coronary stenosis from conventional angiogram^36^. Presence of LGE was interpreted based on the increased signal intensity compared to area of normal myocardium. The visual assessment of LGE has been validated in our earlier study^37^.

As controls in this study, myocardial T1 was measured by placing a region of interest (ROI) in the septum without evidence of first-pass perfusion defects or LGE. Ischemic myocardial T1 was measured by contouring a ROI on T1 maps in an area corresponding to the reversible perfusion defect on first-pass stress CMR but without LGE. Infarcted myocardial T1 was measured by a ROI in the core of the infarcts on LGE images. Remote myocardial ROI were placed contralateral to the ischemic myocardium in areas without evidence of perfusion defects, regional wall motion abnormalities, or LGE. To avoid partial volume contamination from the blood pool, all ROIs were positioned away from the endo- and epicardial borders. The difference in T1 between rest and adenosine stress, i.e. T1 reactivity, was reported in absolute numbers ΔT1 (ms) = T1 adenosine − T1 rest, and as percentages (%) = ΔT1 ÷ T1 rest × 100.

Additional per-segment analysis was performed in the subendocardial region as a blinding process. Each segment was classified as infarct for segment with positive LGE, ischemia for segment with stress induced perfusion defect without infarct, remote ischemia for segment without ischemia in patients with positive ischemia, remote infarct for segment without infarct in patients with positive LGE, and control for segment in patients without ischemia or infarct.

### Statistical analysis

All statistical analyses were performed using SPSS Statistics version 20.0 (SPSS, Inc., Chicago, IL, USA). Continuous data were presented as mean ± standard deviation and compared by the student t-test for unpaired data. Categorical variables are described as number and percentage, and were compared using Fisher’s exact test or chi-square test. ANOVA test with Bonferroni post hoc test was used to compare continuous data from more than 2 groups. Receiver operating characteristic (ROC) curve was used to calculate area under the curve (AUC). A 2-tailed *p* value < 0.05 was considered statistically significant.

## Data Availability

The dataset that was used to support the conclusion of this study is included within the manuscript. Any other additional data will be made available upon reasonable request to the corresponding author.
